# Identification of the source of blood meals in mosquitoes collected from north-eastern Australia

**DOI:** 10.1186/s13071-019-3455-2

**Published:** 2019-05-03

**Authors:** Narayan Gyawali, Andrew W. Taylor-Robinson, Richard S. Bradbury, David W. Huggins, Leon E. Hugo, Kym Lowry, John G. Aaskov

**Affiliations:** 1 School of Health, Medical & Applied Sciences, Central Queensland University, Rockhampton, QLD 4701 Australia; 2 Institute of Health & Biomedical Innovation, Queensland University of Technology, Brisbane, QLD 4059 Australia; 3 Mosquito Control Laboratory, QIMR Berghofer Medical Research Institute, Brisbane, QLD 4006 Australia; 4 School of Health, Medical & Applied Sciences, Central Queensland University, Brisbane, QLD 4000 Australia; 5 Public Environments, Livingstone Shire Council, Yeppoon, QLD 4703 Australia

**Keywords:** Arbovirus, Mosquito, *Culex*, Blood meal, Host, Transmission, Australia

## Abstract

**Background:**

More than 70 arboviruses have been identified in Australia and the transmission cycles of most are poorly understood. While there is an extensive list of arthropods from which these viruses have been recovered, far less is known about the non-human hosts that may be involved in the transmission cycles of these viruses and the relative roles of different mosquito species in cycles of transmission involving different hosts. Some of the highest rates of human infection with zoonotic arboviruses, such as Ross River (RRV) and Barmah Forest (BFV) viruses, occur in coastal regions of north-eastern Australia.

**Methods:**

Engorged mosquitoes collected as a part of routine surveillance using CO_2_-baited light traps in the Rockhampton Region and the adjoining Shire of Livingstone in central Queensland, north-eastern Australia, were analysed for the source of their blood meal. A 457 or 623 nucleotide region of the *cytochrome b* gene in the blood was amplified by PCR and the amplicons sequenced. The origin of the blood was identified by comparing the sequences obtained with those in GenBank®.

**Results:**

The most common hosts for the mosquitoes sampled were domestic cattle (26/54) and wild birds (14/54). Humans (2/54) were an infrequent host for this range of mosquitoes that are known to transmit arboviruses causing human disease, and in an area where infections with human pathogens like RRV and BFV are commonly recorded. The blood meals identified in the most abundant vector analysed, *Culex annulirostris*, were from 10 different vertebrate hosts. The notable detection of chimpanzee blood in two mosquitoes, presumably obtained from a nearby zoo, extends the known range of hosts for this species. *Culex quinquefasciatus* and *Cx. sitiens* fed almost exclusively on a variety of bird species.

**Conclusions:**

While human-mosquito-human transmission of arboviruses like RRV can occur, this study highlights the potential importance of zoonotic cycles of transmission, including avian species, of arboviruses that are indigenous to Australia. Further studies on larger samples of blood-engorged mosquitoes are required to validate the trends observed herein. Moreover, serological and virological evidence that the hosts on which the mosquitoes are feeding are being infected with arboviruses of interest are required.

## Background

Arboviruses (arthropod-borne viruses) are transmitted between hosts by arthropod vectors. While they may infect more than one vertebrate host [1], only some hosts develop viraemias high enough to infect the vectors that feed on them. Australia is home to approximately 380 species of mammals [2], including an estimated 235 species of native marsupials [3]. In addition, 890 species of native and migratory birds have been recorded [4], along with more than 300 species of mosquito [5].

Significant vectors of arboviruses in Australia, including *Aedes camptorhynchus*, *Ae. notoscriptus*, *Ae. vigilax* and *Culex annulirostris*, feed on a range of animal species, including native birds, domestic animals, marsupials and humans [6–11]. However, the composition of animal communities and their proximity to mosquitoes also influence mosquito feeding patterns [12]. In order to determine the health risks associated with individual mosquitoes and to better understand the ecology of mosquito-borne pathogens, it is informative to determine the blood feeding preferences of mosquitoes. Additionally, in order to formulate effective management strategies to control arboviral diseases, it is important to understand the broader ecology and transmission cycles of arboviruses in the areas where this is to happen. A key consideration is what animals are hosts for potential arboviral vectors.

North-eastern Australia is, largely, a tropical region where both mosquito vectors and vertebrate hosts are abundant and in which an ongoing expansion of the human population is predicted [13]. The area chosen for this study is representative of urban and semi-urban areas in north-eastern Australia where several medically relevant mosquito-borne viruses are endemic and acquisition of clinical infections with Ross River (RRV) and Barmah Forest (BFV) viruses is common [14]. The Fitzroy River, one of the longest in Australia, divides the study region from west to east and features a dam that separates tidal salt water downstream from fresh water upstream. Rain and tidal inundation of this river basin contribute to ideal breeding sites for many species of mosquitoes.

## Methods

**Fig. 1:**
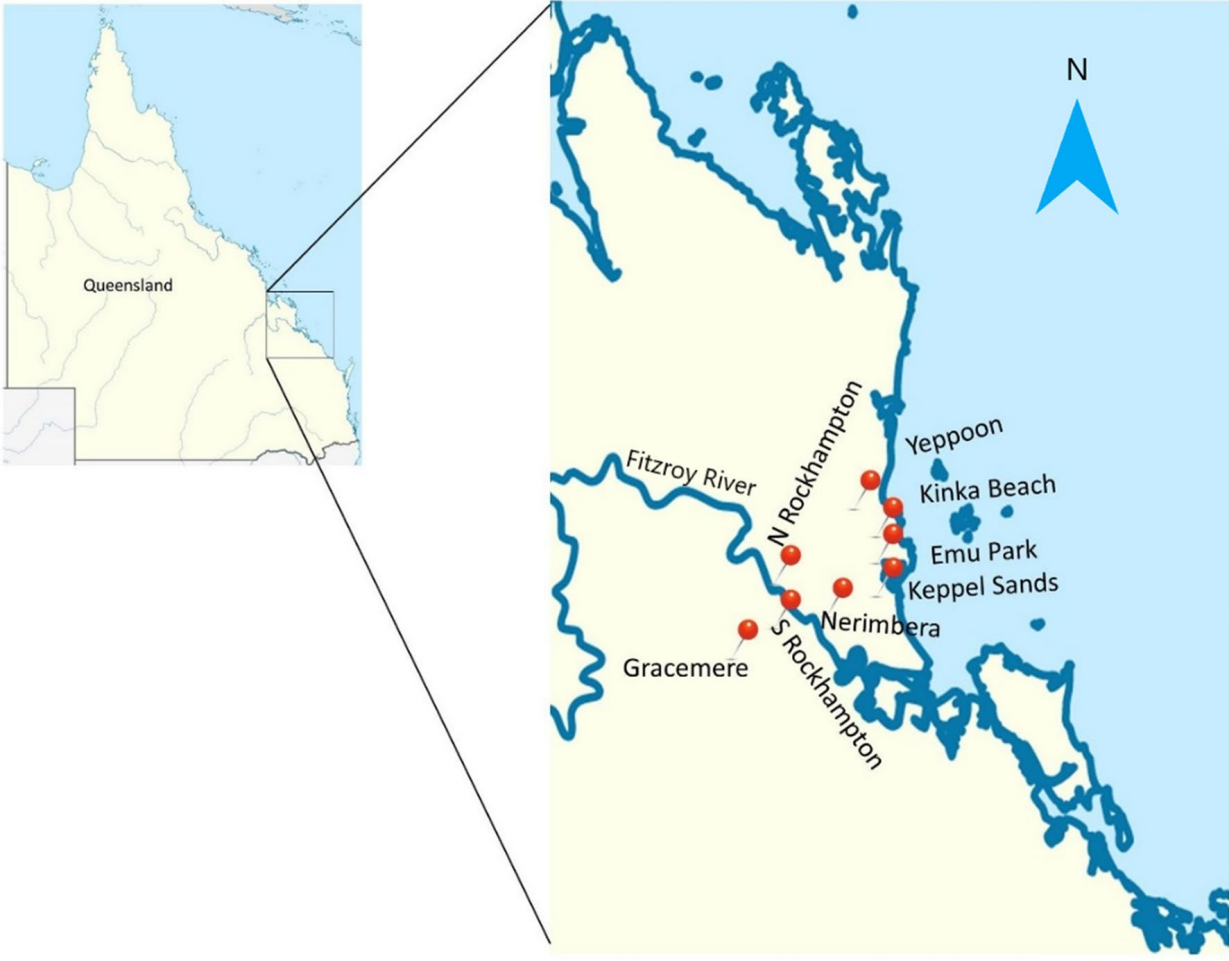
Locations in eastern Central Queensland at which mosquitoes were collected for this study

Mosquitoes were collected in CDC light traps incorporating CO_2_ as a secondary mosquito attractant (CO_2_-baited light trap) [15]. Industrial grade CO_2_ was released at a flow rate of 0.5–1.0 litres/min from gas cylinders (BOC Lindey Group, Rockhampton, QLD, Australia). Collections were carried out at urban and semi-urban localities in the coastal Capricornia region (22°3′3.6″S, 148°11′20.4″E; Fig. 1). Trap sites were positioned in Emu Park, Gracemere, Keppel Sands, Kinka Beach, Nerimbera, North Rockhampton, South Rockhampton and Yeppoon between January and April in 2015 and 2016. Collections were performed as part of the year round routine mosquito control programs undertaken by the Vector Management Unit of the neighbouring Livingstone Shire Council and Rockhampton Regional Council. Mosquito traps were operated overnight and samples collected the following morning. Blood-fed mosquitoes were selected, identified to species and stored at − 80 °C until required for analysis.

A modification of the protocol of Flies et al. (2016) was employed for identification of the mosquito blood meals [16]. Mosquitoes were scanned under a stereo microscope for abdomens that contained blood. These were separated from the head and thorax using a new sterile scalpel blade for each insect and transferred to 2 ml ‘O’ ring screw capped tubes containing three glass beads (0.5 mm each), 180 µl ATL buffer (tissue lysis buffer, Qiagen, Hilden, Germany) and 20 µl proteinase K (Qiagen). The tubes were incubated at 56 °C for 30 min and then were shaken on a MiniBeadbeater™ cell disrupter (BioSpec, Bartlesville, OK, USA). DNA was extracted from the homogenised material using a DNeasy® Blood and Tissue Kit (Qiagen) according to the manufacturer’s instructions. Briefly, 200 µl buffer AL (lysis buffer) was added to the homogenised material, vortexed and incubated at 56 °C for 10 min. Following the incubation, 200 µl ethanol (100%) was added and the mixture was pipetted into a DNeasy Mini spin column (Qiagen) in a 2 ml collection-tube and centrifuged at 6000×*g* for 1 min in a Microfuge®18 (Beckman Coulter, California, USA). Five hundred microlitres of buffer AW1 was added and the spin column centrifuged again for 1 min at 6000×*g*. Buffer AW2 (500 µl) was added to the spin column and centrifuged for 3 min at 20,000×*g*. Between each procedure, the flow-through and collection-tube were discarded and replaced by a new collection tube. DNA was eluted by adding 50 µl elution buffer to the centre of the spin column, followed by centrifugation for 1 min at 6000×*g*. Eluted DNA was stored at − 20 °C.

A segment of the *cytochrome b* gene in the DNA from the blood meal was amplified using 5 µl DNA, 2.5 µl (2 mM) of each forward and reverse primer (Table 1), and 12.5 µl *Taq* PCR master mix (Qiagen) in a final volume of 25 µl. For both primer sets (vertebrate [17] and mammalian [18]) the cycling conditions comprised initial denaturation at 94 °C for 3 min, followed by 34 cycles of 94 °C for 30 s (denaturation), 56 °C for 30 s (annealing), 72 °C for 40 s (extension), and a final extension at 72 °C for 10 min.

**Table 1:** Primers used for the amplification of the *cytochrome b* gene of mitochondrial DNA in mosquito blood meals

Primer	Sequence (5’-3’)	Amplicon length (bp)
VF	GAGGMCAAATATCATTCTGAGG	457
VR	TAGGGCVAGGACTCCTCCTAGT	457
MAMF	TGAGGACAAATATCATTCTGAGG	623
MAMR	GGTTGTCCTCCAATTCATGTTA	623

*Abbreviations*: VF, vertebrate forward; VR, vertebrate reverse; MAMF, mammalian forward; MAMR, mammalian reverse

PCR products (5 µl) were visualised on a 1.5% w/v agarose Tris-acetate/EDTA (TAE) gel stained with SYBR™ Safe Nucleic Acid Staining Solution (ThermoFisher Scientific, Seventeen Mile Rocks, QLD, Australia). Primers, unincorporated dNTPs, enzymes, and salts from the PCR reaction were removed from amplicons using a Purelink® PCR Purification Kit (Invitrogen, Life Technologies, Mulgrave, VIC, Australia) prior to amplicon sequencing. Sanger nucleotide sequencing of the amplified DNA was performed by the Analytical Facility at the QIMR Berghofer Medical Research Institute, Brisbane, QLD, Australia using an Applied Biosystems® 3130 (4-capillary) Genetic Analyzer (ThermoFisher). Sequences were assembled using *de novo* sequence assembly software (version 10) from Geneious (Biomatters Ltd., Auckland, New Zealand) and screened against the NCBI database using the Basic Local Alignment Search Tool (BLAST: https://blast.ncbi.nlm.nih.gov/Blast.cgi). Sample sequences with > 95% identity to sequences in GenBank® were considered a match for the given species [17].

The protocols used for DNA extraction and PCR were validated using horse and dog blood provided by Dr Juliana Chiruta from the School of Biomedical Sciences, Queensland University of Technology and using sheep blood-engorged mosquitoes and non-blood-engorged mosquitoes (colonised *Ae*. *aegypti* from Cairns, QLD, Australia) provided by Dr Gregor Devine, Mosquito Control Laboratory, QIMR Berghofer Medical Research Institute (data not shown).

## Results

A total of 7069 male and female mosquitoes from 20 species were collected in the immediate post-rainfall season months (February to April) of 2015 and 2016. *Culex annulirostris* was the most abundant mosquito trapped (81.9%), followed by *Ae*. *vigilax* (6%). Eighty-one females (1.14%) from six species (Table 2, marked by asterisk) had a discernible red, brown or black mass in a distended abdomen and were selected as, potentially, containing blood.

**Table 2:** Mosquitoes collected in eastern Central Queensland in 2015 and 2016 for this study

Species	No. of specimens	Percentage of total catch (%)
*Aedeomyia catasticta*	39	0.6
*Aedes annulirostris*	200	2.8
*Aedes kochi*	12	0.2
*Aedes lineatopennis*	251	3.6
*Aedes alternans*	2	0.0
*Aedes vigilax	422	6.0
*Aedes vittiger	76	1.1
*Aedes notoscriptus	43	0.6
*Anopheles annularis*	13	0.2
*Coquillettidia xanthogaster*	80	1.1
*Culex australicus*	20	0.3
*Culex annulirostris	5792	81.9
*Culex gelidus*	5	0.1
*Culex quinquefasciatus	11	0.2
*Culex sitiens	42	0.6
*Culex tritaeniorhynchus*	4	0.1
*Mansonia uniformis	36	0.5
*Mimomyia elegans*	1	0.0
*Tripteroides atripes*	9	0.1
*Uranotaenia nivipes*	11	0.2
Total	7069	100

*Blood-fed mosquitoes were recovered from these mosquito species

*Cytochrome b* DNA was amplified from only 10 putative blood-fed mosquitoes following PCR using universal vertebrate or mammalian primers. A second round of PCR using 5 µl amplicon template from the first PCR reaction yielded *cytochrome b* DNA in an additional 53 samples. In 18 of 81 putative blood-fed mosquitoes, no vertebrate or mammalian *cytochrome b* DNA was amplified. Of the remaining 63 abdomens examined, *cytochrome b* DNA was amplified from 16 using vertebrate primers, in 10 using mammalian primers and in 37 abdomens using both vertebrate and mammalian primers.

Sequencing of amplicon DNA including that derived by employing both vertebrate primers and mammalian primers resulted in the identification of the source of blood meals from 54 mosquitoes. These sequences were more than 95% homologous with mammalian or other vertebrate *cytochrome b* gene sequences in GenBank (Table 3). Sequences for *cytochrome b* DNA from two additional blood meals most closely resembled *Centropus bengalensis* (lesser coucal, a species of cuckoo, 91% identity) and *Equus caballus* (horse, 81% identity). However, these values were below the 95% identity proposed as a cut-off [17], so were not included in subsequent analyses. The chromatograms for these two sequences contained multiple peaks at several loci suggestive of blood meals from more than one species. *Cytochrome b* DNA sequences from the remaining seven samples most closely resembled those from species of mosquito. DNA for these particular samples were amplified by mammalian but not by vertebrate primers.

**Table 3:** Sources of blood meals identified in mosquitoes collected in eastern Central Queensland

Common host name	Host species	Mosquito species							Total blood meal sources identified
		*Cx. annuli*	*Cx. quinque*	*Cx. sitiens*	*Man. uniformis*	*Ae. vigilax*	*Ae. vittiger*	*Ae. notoscriptus*	
Human	*Homo sapiens*	2	0	0	0	0	0	0	Mammals (39/54)
Chimpanzee	*Pan troglodytes*	2	0	0	0	0	0	0	
Cattle	*Bos taurus*	23	0	0	0	2	1	0	
Brushtail possum	*Trichosurus vulpecula*	1	0	0	0	0	0	0	
Deer	*Rusa timorensis*	1	0	0	0	0	0	0	
Horse	*Equus caballus*	3	0	0	1	0	0	0	
Pig	*Sus scrofa*	2	0	0	0	0	0	0	
Wallaby	*Macropus agilis*	0	0	0	0	0	0	1	
Carpet python	*Morelia spilota imbricata*	0	0	1	0	0	0	0	Reptile (1/54)
Butcher bird	*Cracticus torquatus*	0	2	1	0	0	0	0	Birds (14/54)
Australian magpie	*Gymnorhina tibicen*	1	1	0	0	0	0	0	
Australian raven	*Corvus coronoides*	2	0	2	0	0	0	0	
Cuckoo	*Centropus bengalensis*	0	1	0	0	0	0	0	
Fig bird	*Sphecotheres vieilloti*	1	0	0	0	0	0	0	
Heron	*Butorides striata*	0	0	2	0	0	0	0	
Pelican	*Pelecanus conspicillatus*	1	0	0	0	0	0	0	
Proportion of mosquitoes with identified blood meal		39/5792 (0.67%)	4/11 (36%)	6/42 (14.2%)	1/36 (2.7%)	2/422 (0.47%)	1/76 (1.3%)	1/43 (2.3%)	

Abbreviations: *Ae*., *Aedes*; *Cx*., *Culex*; *Man*., *Mansonia*; *Cx*., *annuli*, *Culex annulirostris*; *Cx. quinque*, *Culex quinquefasciatus*

The most common hosts for the mosquitoes sampled were domestic cattle (26/54), followed by wild birds (14/54). The source of blood meals identified in 39/54 *Cx. annulirostris* originated from 10 different vertebrate hosts, including a major portion (59%) from cattle. Notably, blood meals from two chimpanzees were identified in *Cx. annulirostris*. The only reptile blood was identified as a blood meal of *Cx. sitiens*, while the other five samples originated from avian species. Similarly, all four blood meals identified in *Cx. quinquefasciatus*, also originated from birds. The locations where the mosquitoes with identified blood meals were collected are indicated in Table 4.

**Table 4:** Locations in eastern Central Queensland where blood-fed mosquitoes were trapped

Mosquito species	Number of mosquitoes with the source of the blood meal identified								
	*Emu Park	*Keppel Sands	*Kinka Beach	*Yeppoon	#Gladstone	#Gracemere	#Nerimbera	#North Rockhampton	#South Rockhampton
*Culex annulirostris*	2	1	1	2	3	5	6	14	5
*Culex quinquefasciatus*	0	0	2	0	0	0	0	0	2
*Culex sitiens*	0	1	0	0	0	0	0	1	4
*Mansonia uniformis*	0	0	0	0	0	1	0	0	0
*Aedes vigilax*	0	0	0	1	0	0	0	1	0
*Aedes vittiger*	0	0	0	0	0	0	0	0	1
*Aedes notoscriptus*	0	0	0	0	0	0	0	1	0
Proportion of mosquitoes with blood meal identified	2/585	2/590	3/1279	3/635	3/405	6/815	6/915	17/844	12/1001
	*10/3089				#44/3980				

*Coastal sites, #Inland sites

## Discussion

Most blood meals in mosquitoes were from animals other than humans despite the collections being undertaken in representative areas of urban and semi-urban human habitations of north-eastern Australia. Given that the region accounts for a large proportion, i.e. ~ 40%, of Australia’s notifications of infections of humans with RRV and BFV [14], the putative hosts of RRV, marsupials (kangaroos and wallabies in particular [19–22]) were expected to be better represented in mosquito blood meals. Hence, the relative absence of blood meals from members of the kangaroo family may reflect the low abundance of these marsupials in the region from which the mosquitoes were collected. Previous studies have found macropod blood in similar mosquito species to those studied here [11, 23] and a high prevalence of antibodies against RRV and other arboviruses in macropods [19–22], indicating that they are being exposed to mosquito-borne viruses. While other trapping methods may have sampled different mosquito species or increased the yield of some of the less well represented species in this collection (80% of which were *Cx. annulirostris*), there were no major omissions of mosquito species that have been implicated in transmission of arboviruses known to cause human disease in this area.

A preference of *Cx. quinquefasciatus* and *Cx. sitiens* for avian hosts (Table 3) has not been reported previously. There is a body of literature that suggests that *Cx. quinquefasciatus* is an opportunistic feeder which acquires blood from a diverse range of birds and mammals, depending upon the relative abundance and availability of vertebrate hosts within a specific geographical area [24–28]. While this provides an insight into vector preferences, the low number of blood-fed mosquitoes collected (reflecting the well-known difficulty of sampling mosquitoes at this life stage due to their preference to rest while digesting blood meals) does not permit a conclusive determination of mosquito host preferences. Earlier studies [9, 11, 23] that identified a significant number of *Cx. annulirostris* containing avian blood meals lacked the technology to identify from which avian species the blood was derived.

Although there is serological evidence of infection of birds with a range of indigenous Australian arboviruses [29], only a limited number of viruses have been recovered from them: RRV from the Australian magpie (*Grallina cyanoleuca*), jacky winter (*Microeca fascinans*) and masked finch (*Poephila personata*); Kunjin virus from the green oriole (*Oriolus flavocinctus*) and Alfuy virus from the coucal (*Centropus phasianus*) [30]. Since a number of Australian arboviruses have been isolated from *Cx. quinquefasciatus* [31, 32] and *Cx. sitiens* [33], it is possible that these arboviruses may have been maintained in transmission cycles which involve birds as hosts and *Cx. quinquefasciatus* or *Cx. sitiens* as vectors. A novel observation in the present study was blood originating from a carpet python (*Morelia spilota*) in a *Cx. sitiens* mosquito, although there are reports of other *Culex* species feeding on snakes in the USA [34].

Blood meals from approximately 80% of these mosquitoes were from mammals, predominantly cattle (Table 3). A range of vertebrates, including humans, horses, cattle, dogs, marsupials and birds, have been suggested as hosts for *Cx. annulirostris* (Table 5), depending on the accessibility of each. The high proportion of blood meals from cattle but not from either small domestic pets (cats or dogs) or native animals (e.g. marsupials) may reflect the greater accessibility of these large hosts to the mosquitoes, especially as cattle farming is a leading industry in eastern Central Queensland. As *Cx. annulirostris* has a flight range of up to 7 km [35, 36], those specimens sampled in this study may have fed on hosts some considerable distance from where they were trapped. Current observations suggesting a potential role for cattle in the ecology of Australian arboviruses are supported by serological evidence of bovine infection with arboviruses [37–42], and also by the isolation of arboviruses from *Cx. annulirostris* [10, 43–45].

**Table 5:** Mosquito vectors and vertebrate hosts revealed by previous Australian mosquito blood-meal analysis studies

Reference	Locations of mosquitoes sampled	Method of detection	Most abundant blood-fed vector(s)	Vertebrate host
Kay et al. [9]	Brisbane and Carseldine (outer suburban Brisbane)	Agar gel immunodiffusion	*Cx. annulirostris*	Common brushtail possums, horses, dogs, humans and birds
Kay et al. [23]	Mitchell River Mission and Charleville	Precipitin test	*Cx. quinquefasciatus*; *Cx. annulirostris*	Dogs, macropods, cattle, pigs, humans and birds
van den Hurk et al. [11]	Far North Queensland	Agar gel immunodiffusion	*Cx. annulirostris*	Mammals, marsupials, pigs and birds
Jansen et al. [7]	Urban and peri-urban habitats in eastern Australia (Brisbane, Cairns, Newcastle and Sydney)	Enzyme-linked immunosorbent assay	*Ae. aegypti*; *Cx. annulirostris*	*Ae. aegypti* had predominantly human blood; *Cx. annulirostris* had cattle, marsupial, dog and human blood
Johansen et al. [8]	Western Australia	Enzyme-linked immunosorbent assay	*Ae. camptorhynchus*; *Cx. annulirostris*	Marsupials and cattle

The promiscuous feeding habits of *Cx. annulirostris* were exemplified by the detection of non-human primate blood in two mosquitoes. As there are no non-human primate species native to Australia, these were almost certainly derived from the five common chimpanzees (*Pan troglodytes*) held at the Rockhampton zoo, which houses the only chimpanzees in Queensland. The closest collection site was approximately 800 metres from the zoo.

Identification, by PCR and sequencing, of mitochondrial *cytochrome b* DNA from vertebrates in mosquito blood meals is more sensitive and specific than employing enzyme linked immunosorbent assays or gel diffusion for this purpose [46]. There was a lower rate of blood meal identification in this study (~ 70%) than in some other investigations employing molecular techniques where identification rates from 80 to 100% were reported [16, 47]. However, reports of high identification rates usually employed only fully engorged insects whereas this study utilised all insects whose abdomen appeared to contain blood of any quantity.

Mosquito collections employing CO_2_-baited light traps selectively attract unfed and partially blood-fed mosquitoes while fully blood-fed mosquitoes prefer to rest and thus are less attracted to CO_2_-baited light traps [48]. All mosquitoes studied in this project were supplied by local authority entomologists and these traps are the tool of choice in the hands of local government authorities. Typically, mosquito traps were operated overnight and insects collected the next morning, a method that, it is acknowledged, may have failed to collect diurnal and crepuscular mosquito species. Since *Ae. aegypti* is diurnal, this species is very rarely collected from CO_2_-baited light traps [49] but this species is extremely rare in the area of this study. This bias might have been minimised if it had been possible to trap mosquitoes over 24 hours using different mosquito traps and alternative methodologies [50, 51]. Targeted approaches to collecting blood-fed mosquitoes, e.g. small resting boxes such as those used by Brugman et al., 2017 [52] or large resting traps used by Sandhu et al., 2013 [53], may have yielded greater numbers of blood-fed mosquitoes and should be considered in any future studies.

## Conclusions

While human-mosquito-human transmission of arboviruses like RRV can occur, this study highlights the potential importance of zoonotic cycles to the transmission of Australian arboviruses. In turn, this stresses how important it is that vector/disease control programs should be informed by an accurate knowledge of local hosts and vectors. There is sufficient novelty in the results of this study to justify the use of additional tools or techniques in a more targeted approach to a sizeable collection of blood-fed mosquitoes and the identification of their blood meal by local authorities. The low catch rates for the target group, blood-fed mosquitoes, did not enable definitive conclusions about mosquito host preferences to be drawn. However, a relatively high proportion of avian blood meals in the mosquitoes studied provides a rationale for focussing attention on wild birds as possible reservoir/amplifying hosts for Australian arboviruses.

## Abbreviations

BFV: Barmah Forest virus
CDC: Centers for Disease Control and Prevention
PCR: Polymerase Chain Reaction
QLD: Queensland
RRV: Ross River virus
VIC: Victoria
